# Native hyphal alignment enables spatially anisotropic functions in *Pleurotus eryngii*-derived membranes for guided bone regeneration

**DOI:** 10.3389/fcell.2026.1997731

**Published:** 2026-10-23

**Authors:** Xiao Chen, Nihang Dong, Hanbo Hou, Yang Li, Haipeng He, Jiamin Huang, Hongwen Li, Jiang Chen, Quanli Li

**Affiliations:** 1 Department of Stomatology, Institute of Oral Science, Shenzhen Longgang Otolaryngology Hospital & Shenzhen Otolaryngology Research Institute, Shenzhen, China; 2 Center for Intelligent Biomedical Materials and Devices (BMD), Shenzhen Institutes of Advanced Technology (SIAT), Chinese Academy of Sciences (CAS), Shenzhen, China; 3 Key Laboratory of Oral Diseases Research of Anhui Province, College & Hospital of Stomatology, Anhui Medical University, Hefei, China; 4 The Department of Ophthalmology, Sichuan Provincial People’s Hospital, University of Electronic Science and Technology of China, Chengdu, China; 5 Translational Clinical Immunology Key Laboratory of Sichuan Province, Sichuan Provincial People’s Hospital, University of Electronic Science and Technology of China, Chengdu, China

**Keywords:** contact guidance, directional slicing, functional anisotropy, guided bone regeneration, native hyphal architecture, *Pleurotus eryngii*

## Abstract

Guided bone regeneration (GBR) membranes are required to coordinate mechanical support, osteogenic cell regulation, and soft-tissue exclusion across different spatial directions. Hierarchical architectures with spatial heterogeneity and anisotropy may therefore better accommodate these direction-dependent functional requirements. However, bottom-up construction of such architectures often relies on specialized equipment and complex multistep fabrication. By contrast, biological tissues spontaneously develop intricate heterogeneous and hierarchical structures during growth, providing unique value for the top-down construction of architecture-driven biomaterials. The widely cultivated *Pleurotus eryngii* has hyphal cell walls composed mainly of chitin and β-glucan, while its fruiting-body stipe exhibits a pronounced longitudinal organization and a robust morphology amenable to sectioning, making it suitable for generating distinct structural presentations simply by changing the cutting orientation. In this study, cross-cut (Tr-C) and longitudinal-cut (Tr-L) membranes were prepared from *P. eryngii* stipes using the same combined chemical-enzymatic treatment, which substantially reduced cell-associated residues while preserving recognizable tubular hyphal architecture. Tr-C and Tr-L exhibited similar overall chemical characteristics, yet the structural differences introduced by cutting orientation resulted in clearly distinct functional states. Tr-L showed more pronounced directional wet-state mechanical behavior, stronger contact guidance of MC3T3-E1 cells, enhanced osteogenesis-related phenotypes, and greater fibroblast exclusion across the membrane thickness. In contrast, Tr-C exhibited faster liquid infiltration and greater hydration capacity. The two membrane architectures also induced distinct macrophage response patterns under inflammatory stimulation. In a rat calvarial defect model, Tr-L promoted more pronounced centripetal new bone formation. These findings demonstrate that different structural presentations of the same native hyphal network can generate membrane-scale functional anisotropy, highlighting the value of native hyphal organization for constructing GBR membranes with spatially differentiated functions.

## 1 Introduction

Guided bone regeneration (GBR) is a widely used bone augmentation technique in oral implantology and maxillofacial bone defect repair. Its fundamental principle is to interpose a barrier membrane between the bone defect and rapidly proliferating soft tissues, thereby maintaining a stable regenerative space and blood clot while creating a favorable environment for the migration of osteogenic cells and new bone formation (Donos et al., 2023). Accordingly, an ideal GBR membrane should not only exhibit good biocompatibility and effective soft-tissue exclusion, but also provide sufficient wet-state mechanical stability, appropriate fluid and nutrient transport, and a degradation profile compatible with the tissue repair process (Mizraji et al., 2023). Importantly, these functional requirements are not spatially uniform. The bone-facing surface and in-plane architecture should support the adhesion, migration, and organization of osteogenic cells, whereas the through-thickness direction should effectively restrict the infiltration of fibroblasts and other soft-tissue cells into the defect. Recent GBR materials with layered, asymmetric, or directionally porous architectures have sought to spatially coordinate cell ingress and soft-tissue exclusion, further highlighting the importance of spatially organized structure-function relationships in GBR membrane design (Wu et al., 2024; Jiang et al., 2022).

Beyond biochemical signals arising from material composition, the mechanical properties, surface topography, and three-dimensional geometry of a biomaterial can themselves act as persistent biophysical cues that regulate cell adhesion, spreading, migration, orientation, and differentiation (Li et al., 2017). Unlike diffusible biochemical factors, many structure-derived biophysical cues are spatially confined to the cell-material interface and are therefore largely contact-dependent. This spatial confinement allows persistent and localized regulation of cell behavior without relying on sustained molecular release, although it may also limit the effective range of such signals beyond the immediate material interface. In particular, contact guidance provided by aligned fibers, microgrooves, and ordered porous structures can reorganize the cytoskeleton and alter cell morphology and alignment. In osteogenic cells, microgrooved surfaces have been shown to markedly regulate MC3T3-E1 cell elongation, orientation, focal adhesion formation, and F-actin organization without substantially altering cell proliferation, while also enhancing osteogenic differentiation and new bone formation *in vivo* (Zhu et al., 2019). Similarly, three-dimensional scaffolds with radial or other directionally organized hierarchical architectures can guide bone marrow-derived mesenchymal stem cells (BMSCs) along predefined migration paths and further influence the spatial distribution and organization of regenerated bone (Chen et al., 2021). However, artificial approaches such as electrospinning, micro-/nanopatterning, directional freezing, and three-dimensional printing generally require specialized equipment and multiple fabrication steps. By contrast, natural biological tissues already possess complex hierarchical structures and intrinsic directionality developed during growth. Preserving and directly utilizing such native architectures may therefore offer a simpler route to constructing architecture-driven biomaterials.

The stipe of *Pleurotus eryngii* possesses pronounced native hierarchical anisotropy. Its tissue is composed of interconnected tubular hyphal units, whose cell walls are mainly built from structural polysaccharides such as chitin and glucans that collectively contribute to mechanical support (Jones et al., 2021; Sun et al., 2022). Recent structural analyses of minimally reconstructed *P. eryngii* stipes further showed that micron-scale hyphae are preferentially organized along the longitudinal axis, resulting in distinctly different microstructural features between transverse and longitudinal sections (Matsuura et al., 2026). This native architecture has already been exploited in top-down material fabrication, although the extent to which it is preserved strongly depends on the processing conditions. For example, intensive or multistep chemical treatments can retain the overall sponge-like form of *P. eryngii* while substantially remodeling the original hyphal microstructure into reconstructed porous networks with enlarged pores and increased interconnectivity (Fang et al., 2024). In contrast, optimized and relatively mild decellularization procedures can better preserve the fibrous and anisotropic microarchitecture of mushroom tissues, allowing the retained directional features to further guide cellular alignment (Kim et al., 2025). More recently, directional mushroom-derived scaffolds have exploited the intrinsic aligned architecture of mushroom stipes to guide BMSC migration and enhance bone defect repair, further demonstrating the regenerative potential of native mushroom anisotropy (Yang et al., 2025).

Nevertheless, previous studies have generally treated the intrinsic directionality of mushroom tissues as a predefined property of three-dimensional scaffolds to be preserved and utilized. How different cutting planes alter the geometric presentation of a well-preserved tubular hyphal network within a two-dimensional membrane, and whether such structural translation can further generate distinct membrane-scale functions, remain insufficiently understood. For *P. eryngii* stipes with a clear longitudinal organization, longitudinal cutting exposes more tubular hyphal units extending along the membrane plane, whereas cutting perpendicular to the stipe axis exposes more transverse and oblique hyphal profiles together with wall structures of different orientations. Thus, even with a similar material origin and overall chemical background, changing the cutting direction alone may reorganize load-transfer pathways, fluid-transport routes, cell-perceivable surface topography, and through-thickness migration barriers, thereby generating distinct physical and biological functions. The key concept is therefore not to create a new anisotropy, but rather to use the cutting plane to reinterpret structural information already encoded in the native hyphal architecture.

Based on this concept, *P. eryngii* stipes were used as natural structural templates and sectioned in either the cross-cut or longitudinal-cut direction to obtain membrane-like specimens with different structural presentations. Both types of specimens were subsequently subjected to the same combined chemical-enzymatic treatment to substantially reduce residual DNA and protein while preserving recognizable tubular hyphal architecture as much as possible. We hypothesized that directional slicing could translate the intrinsic structural information of the same native three-dimensional hyphal network into membrane-scale functional anisotropy by altering how the network is presented within the membrane. To test this hypothesis, treated cross-cut (Tr-C) and longitudinal-cut (Tr-L) membranes were systematically compared in terms of microstructure, chemical characteristics, wet-state mechanics, fluid infiltration, water uptake, and *in vitro* mass changes. Their effects on macrophage inflammatory responses, osteogenic cell morphology and orientation, osteogenesis-related phenotypes, and fibroblast transmembrane migration were further evaluated, followed by assessment of *in vivo* bone regeneration in a rat calvarial defect model. Through these analyses, this study aimed to establish a structure-function relationship linking native hyphal architecture, cutting orientation, functional anisotropy, cellular behavior, and bone regeneration, and to explore a simple GBR membrane design strategy that uses native hierarchical architecture to simultaneously provide in-plane cell guidance and through-thickness soft-tissue exclusion.

## 2 Materials and methods

### 2.1 Preparation of *Pleurotus eryngii*-derived membrane specimens

Fresh *Pleurotus eryngii* mushrooms were purchased from a local Yonghui Supermarket. The middle region of morphologically intact and relatively homogeneous stipes was selected for specimen preparation. Tissue slices with a thickness of approximately 1 mm were cut either parallel or perpendicular to the longitudinal axis of the stipe to obtain untreated longitudinal-cut specimens (Un-L) and untreated cross-cut specimens (Un-C), respectively.

Both types of specimens were subjected to the same combined chemical-enzymatic treatment. First, the specimens were completely immersed in a solution containing 2.5% (w/v) Na_2_CO_3_ and 0.25% (w/v) sodium dodecyl sulfate (SDS) and treated at 60 °C and 80 rpm for 2 h. The specimens were then thoroughly washed with deionized water at a wet specimen-to-water ratio of approximately 1 g:25 mL. Each washing cycle was performed at 37 °C and 80 rpm for 2 h using freshly replaced deionized water, for at least five cycles.

The washed specimens were subsequently immersed in a 10 U/mL β-glucanase solution (Shanghai Yuanye Bio-Technology Co., Ltd., China) adjusted to pH 5.5, at a wet specimen-to-enzyme solution ratio of approximately 1 g:25 mL. Enzymatic treatment was performed at 50 °C and 80 rpm for 12 h and repeated once using freshly prepared enzyme solution. After enzymatic treatment, the specimens were again washed with deionized water for at least five cycles under the conditions described above. Following the final washing step, the specimens were treated in a 90 °C water bath for 30 min.

The treated specimens were rapidly frozen on an aluminum tray pre-cooled to −20 °C to minimize ice-crystal-induced damage to the hyphal architecture, and were subsequently lyophilized at −80 °C under a vacuum of 0.37 Pa. The resulting treated cross-cut and longitudinal-cut specimens were designated Tr-C and Tr-L, respectively, and stored under dry conditions until further use.

### 2.2 Gross morphology and scanning electron microscopy

The gross morphology of freeze-dried Un-C, Un-L, Tr-C, and Tr-L specimens was first recorded by photography. Representative specimens were then examined using scanning electron microscopy (SEM; Sigma 360, ZEISS, Germany). Before imaging, the samples were sputter-coated with platinum. SEM images acquired at different magnifications were used to examine the overall hyphal network morphology, hyphal orientation, and deposits on the outer and luminal surfaces of the tubular hyphal structures.

### 2.3 Histological observation and quantification of residual DNA and protein

To evaluate the internal architecture and residual cellular components before and after treatment, Un-C, Un-L, Tr-C, and Tr-L specimens were fixed in 4% paraformaldehyde, dehydrated through a graded ethanol series, embedded in paraffin, and sectioned at a thickness of approximately 3 μm along the corresponding observation planes. The sections were stained with hematoxylin and eosin (H&E) and examined by optical microscopy to assess internal hyphal architecture and staining characteristics.

Residual DNA was extracted using a Fungi Genomic DNA Extraction Kit (Macklin, China) and quantified using PicoGreen (Shanghai Yuanye Bio-Technology Co., Ltd., China). DNA content was normalized to the dry mass of the specimens and expressed as ng/mg. Residual protein was extracted using a Fungi Protein Extraction Kit (Beijing Solarbio Science & Technology Co., Ltd., China) and quantified using a bicinchoninic acid (BCA) assay kit (Beyotime Biotechnology, China). Protein content was likewise normalized to the dry mass of the specimens and expressed as μg/mg.

### 2.4 ATR-FTIR analysis

The overall chemical characteristics of Un-C, Un-L, Tr-C, and Tr-L specimens were analyzed by attenuated total reflectance Fourier-transform infrared spectroscopy (ATR-FTIR; Nicolet iN10, Thermo Fisher Scientific, USA). β-Glucan and chitin reference samples were analyzed under the same conditions for comparison.

Spectra were collected over the range of 4000–800 cm^−1^ at a spectral resolution of 0.5 cm^−1^, with 32 accumulated scans for each specimen. All spectra were processed using the same baseline correction and normalization procedures.

### 2.5 Wet-state uniaxial tensile testing

The wet-state mechanical properties of Tr-C and Tr-L were evaluated by uniaxial tensile testing using a universal testing machine (CMT4104, Shandong Meister Industrial Testing System Co., Ltd., China). Before testing, the specimens were fully hydrated in PBS at 37 °C.

For Tr-L, tensile loading was applied either parallel or perpendicular to the principal hyphal orientation, designated Tr-L (II) and Tr-L(T), respectively. Tr-C specimens were loaded along the longitudinal axis of the tensile specimen. The specimens had a width of 10 mm, an initial gauge length of 15 mm, and a thickness of 0.3 mm. Tensile testing was performed at a crosshead speed of 1 mm/min.

Engineering stress was calculated from the applied force and the initial cross-sectional area of the specimen. Tensile strength was defined as the maximum tensile stress recorded during testing, and the elastic modulus was determined by linear fitting of the initial approximately linear region of the engineering stress-strain curve using the instrument software.

### 2.6 Liquid infiltration and hydration

#### 2.6.1 Dynamic liquid infiltration

Tr-C, Tr-L (II), and Tr-L(T) membranes were vertically fixed with their lower edges brought into simultaneous contact with α-MEM supplemented with 10% fetal bovine serum. For Tr-L(II) and Tr-L(T), liquid migration occurred parallel and perpendicular to the principal hyphal orientation, respectively. Representative images were collected at 0, 1, 5, 10, and 15 s.

#### 2.6.2 Hydrated-to-dry mass ratio

Freeze-dried membranes were weighed to obtain the initial dry mass ( $W_{d}$ ) and then immersed in deionized water until fully hydrated. After removal from the solution, excess surface water was gently blotted with filter paper, and the wet mass ( $W_{w}$ ) was immediately recorded. The hydrated-to-dry mass ratio was calculated as:

$$Q=\frac{W_{w}}{W_{d}}$$

where $W_{d}$ is the initial dry mass and $W_{w}$ is the fully hydrated mass. The ratio was expressed as g/g.

### 2.7 *In vitro* mass change

Freeze-dried Tr-C and Tr-L specimens were separately incubated in three media: phosphate-buffered saline (PBS), PBS containing 50 μg/mL lysozyme, and PBS containing 200 μM H_2_O_2_. All media were additionally supplemented with 1% Penicillin-Streptomycin-Amphotericin B Solution (100×; Beyotime Biotechnology, China). The specimen-to-medium ratio was approximately 5 mg/mL, and the samples were incubated at 37 °C for 4 weeks.

The incubation media were completely replaced with freshly prepared solutions every day. Accordingly, the nominal H_2_O_2_ concentration of 200 μM refers to the initial concentration after each medium replacement rather than a constant concentration maintained throughout the subsequent 24 h.

At predetermined time points, the specimens were collected, thoroughly rinsed with deionized water, lyophilized, and weighed. The remaining mass was calculated as:

$$\text{Mass remaining}\left(\%\right)=\frac{W_{t}}{W_{0}}\times 100\%$$

where $W_{0}$ is the initial dry mass before incubation and $W_{t}$ is the dry mass at the corresponding time point.

Because small fragments detached during incubation were not recovered, the measured mass change was used to characterize apparent mass loss/*in vitro* erosion rather than a direct measure of specific chemical degradation.

### 2.8 RAW264.7 macrophage culture and evaluation of inflammatory responses

RAW264.7 murine macrophages were obtained from BaiDi Biotechnology Co., Ltd (BDBIO, China) and cultured in DMEM supplemented with 10% fetal bovine serum (FBS) and 1% penicillin-streptomycin at 37 °C in a humidified atmosphere containing 5% CO_2_. Tr-C and Tr-L specimens were sterilized by ultraviolet irradiation and placed in 48-well plates. RAW264.7 cells were seeded onto the Control, Tr-C, and Tr-L substrates at a density of 4 × 10^4^ cells/well. Experiments were performed under either unstimulated [LPS(−)] or lipopolysaccharide-stimulated [LPS(+)] conditions, with a final LPS concentration of 100 ng/mL.

#### 2.8.1 iNOS immunofluorescence staining

After 24 h of culture, the cells were fixed with 4% paraformaldehyde and subsequently treated as follows: (1) incubation with PBST for 20 min at room temperature; (2) treatment with PBS containing 100 mM glycine for 10 min; (3) two washes with PBST for 5 min each; (4) permeabilization with PBS containing 0.1% Triton X-100 for 20 min; (5) three washes with PBST for 5 min each; and (6) blocking with PBST containing 5% bovine serum albumin (BSA; Aladdin, China) for 2 h.

The samples were then incubated with CoraLite®594-conjugated iNOS Polyclonal Antibody (Proteintech, Wuhan, China) at 4 °C in the dark for 12 h, followed by six 10-min washes with PBST at room temperature in the dark. Cell nuclei were counterstained with DAPI, followed by three 5-min washes with PBS. Fluorescence images were acquired using a confocal laser scanning microscope (LSM 910, ZEISS). Because Tr-C and Tr-L exhibited substantial intrinsic substrate fluorescence, the iNOS images were used primarily for qualitative assessment, and fluorescence intensity within the material regions was not directly quantified.

#### 2.8.2 CCK-8 assay

Cell metabolic activity was evaluated after 24 and 72 h of culture using a Cell Counting Kit-8 (CCK-8; Beyotime Biotechnology, China). Cells were incubated with CCK-8 reagent according to the manufacturer’s instructions. After incubation, the reaction solution was transferred to a new 96-well plate, and absorbance was measured at 450 nm to minimize optical interference from the porous mushroom-derived membranes.

#### 2.8.3 TNF-α measurement

After 24 h of culture, the culture supernatants were collected, and TNF-α concentrations were determined using a mouse TNF-α ELISA kit (CUSABIO, Wuhan Huamei Bioengineering Co., Ltd., China) according to the manufacturer’s instructions.

Because the porous mushroom-derived membranes interfered with conventional total-protein quantification, TNF-α levels were not normalized to BCA-derived total protein. Instead, CCK-8 measurements obtained in parallel were used to determine whether differences in inflammatory output were accompanied by marked differences in cellular metabolic activity.

#### 2.8.4 Griess assay

After 72 h of culture, the supernatants were collected and nitrite concentrations were measured using a Griess-based Nitric Oxide Assay Kit (Beyotime Biotechnology, China) as an indicator of NO-related inflammatory output. Nitrite concentrations were calculated from the corresponding standard curve. Four independent samples were used for each experimental condition.

### 2.9 MC3T3-E1 cell culture and cytocompatibility

MC3T3-E1 murine preosteoblasts were obtained from BaiDi Biotechnology Co., Ltd (BDBIO, China) and cultured in α-MEM (Gibco, USA) supplemented with 10% FBS (Gibco, USA) and 1% penicillin-streptomycin (Beyotime Biotechnology, China) at 37 °C in a humidified atmosphere containing 5% CO_2_.

Sterilized Tr-C and Tr-L specimens were placed in 48-well plates, with cell culture coverslips used as the Control. MC3T3-E1 cells were seeded at 1 × 10^4^ cells/well. Cell metabolic activity was evaluated after 1, 3, and 5 days using the CCK-8 assay as described above.

### 2.10 MC3T3-E1 cell morphology and orientation analysis

After 1 day of culture, F-actin staining was performed to evaluate cell morphology and orientation. Samples were fixed with 4% paraformaldehyde and washed with PBS. F-actin was stained using a ready-to-use fluorescent phalloidin reagent (Beyotime Biotechnology, China), and cell nuclei were counterstained with DAPI (Beyotime Biotechnology, China). Images were acquired using a confocal laser scanning microscope (LSM 910, ZEISS). The green signal observed in Tr-C and Tr-L images originated from intrinsic autofluorescence of the mushroom-derived substrates.

#### 2.10.1 Substrate orientation analysis

The Directionality plugin in Fiji was used to analyze the orientation distributions of the Control, Tr-C, and Tr-L substrates. Because structural orientation is axial, with 0° and 180° representing the same direction, the axial orientation order parameter ( R ) was calculated using a double-angle approach.

$$C=\frac{\sum w_{i}\cos2\theta_{i}}{\sum w_{i}}$$

$$S=\frac{\sum w_{i}\sin2\theta_{i}}{\sum w_{i}}$$

$$R=\sqrt{C^{2}+S^{2}}$$

where $\theta_{i}$ represents the structural orientation angle and $w_{i}$ represents the corresponding frequency weight. R ranges from 0 to 1, with values approaching 0 indicating a largely random orientation and values approaching 1 indicating a highly aligned structure.

#### 2.10.2 Cell morphology and orientation analysis

F-actin images were processed using a consistent thresholding procedure in Fiji. The Analyze Particles and Fit Ellipse functions were used to determine the major axis, minor axis, and orientation angle of individual cells. Cell aspect ratio was calculated as:

$$\text{Aspect ratio}=\frac{\text{Major}\;\text{axis}}{\text{Minor}\;\text{axis}}$$

For group-level statistical comparisons of cell morphology and orientation, each independent specimen was treated as one experimental unit; individual cells within a specimen were not treated as independent replicates. Single-cell data were used only for descriptive analysis of the relationship between Δθ and cell aspect ratio.

The cellular orientation order parameter was calculated using the same double-angle method described for substrate orientation. The minimum axial deviation angle ( Δθ ) between the major axis of each cell and the principal orientation of the corresponding substrate was further calculated within a range of 0–90°. The relationship between Δθ and cell aspect ratio was then analyzed.

### 2.11 Osteogenic induction and ALP assay

MC3T3-E1 cells cultured on the Control, Tr-C, and Tr-L substrates were subjected to osteogenic induction. The osteogenic medium consisted of α-MEM supplemented with 10% FBS, 1% penicillin-streptomycin, 10 mM β-glycerophosphate, 50 μg/mL ascorbic acid, and 100 nM dexamethasone. The culture medium was replaced every 2 days.

After 14 days of osteogenic induction, the samples were washed with PBS and fixed, followed by alkaline phosphatase (ALP) staining using a BCIP/NBT Alkaline Phosphatase Color Development Kit (Beyotime Biotechnology, China) according to the manufacturer’s instructions. Representative images were subsequently acquired.

ALP activity was quantified in parallel using an ALP assay kit (Beyotime Biotechnology, China). The dsDNA content of the corresponding samples was quantified using a PicoGreen assay with Lamda DNA (MCE, China) as the standard. Because the mushroom-derived membranes contained residual DNA-associated signals, the contribution of substrate-derived DNA was estimated based on the residual DNA content determined from acellular membranes using the method described in Section 2.3. The calculated substrate-derived DNA background was subtracted from the total measured DNA content, and ALP activity was normalized to the corrected dsDNA content and expressed as U/mg DNA.

### 2.12 Alizarin red S staining and quantification

After 21 days of osteogenic induction, Alizarin Red S (ARS) staining was performed using an Alizarin Red S Staining Quantification Kit based on the cetylpyridinium chloride (CPC) method (Beijing Solarbio Science & Technology Co., Ltd., China).

The samples were washed with PBS and fixed with 4% paraformaldehyde. Staining was performed according to the manufacturer’s instructions, followed by thorough washing with distilled water to remove unbound dye. Representative gross and microscopic images were then acquired.

The bound ARS was subsequently eluted and extracted according to the kit protocol, and the absorbance of the extracted solution was measured at 560 nm to quantify mineralization-associated staining.

### 2.13 Transwell assay for fibroblast transmembrane migration

The cell-barrier capacity of Tr-C and Tr-L membranes was evaluated using 12-well Transwell inserts with an 8-μm pore size (BIOFIL, JET Life Science (Guangzhou) Co., Ltd., China). Tr-C and Tr-L membranes were placed in the upper chambers to completely cover the porous Transwell membrane. The membrane edges were positioned in close contact with the insert to minimize cell migration around the specimen edges. Uncovered Transwell membranes served as the Control.

L929 fibroblasts were seeded onto the upper surface of the membranes at a density of 3 × 10^4^ cells/well. Culture medium containing 10% FBS was added to the upper chamber, while medium containing 20% FBS was added to the lower chamber to establish a serum gradient.

After 5 days of culture, non-migrated cells on the upper surface were removed, and cells that had migrated to the lower surface of the Transwell membrane were stained with crystal violet. Multiple fields were randomly imaged at a fixed magnification, and migrated cells were counted using ImageJ. The results were expressed as the number of migrated cells per unit area.

### 2.14 Rat calvarial defect model

All animal procedures were approved by the Ethics Review Committee of Shenzhen Longgang ENT Hospital on Laboratory Animal Care (No. 2026-0230) and were conducted in accordance with the relevant guidelines for animal experimentation.

Male Sprague-Dawley (SD) rats, approximately 3 months old and weighing >300 g, were used. The animals were anesthetized with inhaled isoflurane. After shaving and disinfection of the calvarial region, a midline scalp incision was made to expose the calvarium. The periosteum over the defect area was removed, and two bilateral full-thickness circular defects, each 5 mm in diameter, were created using a trephine bur under continuous saline irrigation. Care was taken to avoid damage to the underlying dura mater, which was kept intact throughout the procedure.

The present study analyzed defects assigned to the Control, Tr-C, and Tr-L conditions from a larger bilateral calvarial defect experimental cohort. No material was implanted in the Control defects, whereas Tr-C or Tr-L membranes were placed over the corresponding defects in the treatment groups. The same treatment condition was not applied to both defects within the same animal.

Animals were sacrificed at 6 and 12 weeks after surgery. For each treatment group at each time point, four defects were included, with each defect derived from a different animal (n = 4 defects from 4 different animals per group at each time point). The harvested calvarial specimens were fixed in 4% paraformaldehyde for subsequent micro-computed tomography and histological analyses.

### 2.15 Micro-CT analysis

Fixed calvarial specimens were scanned by micro-computed tomography (micro-CT; NMC-200, NEMO) at Wuhan Servicebio Technology Co., Ltd. The scanning speed was 4 s/bed, with a minimum reconstructed pixel size of 2 μm and a spatial resolution of <7.5 μm at 10% MTF.

The original 5-mm-diameter defect region was defined as the region of interest (ROI) for all samples. Three-dimensional reconstruction and quantitative analysis were performed using Recon software.

### 2.16 Histological evaluation

Following micro-CT scanning, the calvarial specimens were decalcified in EDTA, dehydrated through a graded ethanol series, embedded in paraffin, and sectioned through the central region of the defect at a thickness of 3 μm.

Serial sections were subjected to hematoxylin and eosin (H&E) staining and Masson’s trichrome staining. Low-magnification panoramic images and corresponding high-magnification images were acquired using a digital slide scanner. Bone regeneration within the defect area was evaluated according to the morphological characteristics and distribution of old bone (OB) and newly formed bone (NB).

### 2.17 Statistical analysis

All quantitative data are presented as mean ± standard deviation (SD). Statistical analyses were performed using GraphPad Prism 8. One-way or two-way ANOVA, as appropriate, followed by Tukey’s multiple comparisons test, was used for group comparisons. Unless otherwise specified, differences were considered statistically significant at p<0.05 .

## 3 Results and discussion

### 3.1 Preparation and structural characterization of *Pleurotus eryngii*-derived membranes

As illustrated in Figure 1A *P. eryngii* stipes were sectioned in the cross-cut and longitudinal-cut directions to obtain Un-C and Un-L specimens, respectively. After the combined chemical-enzymatic treatment, washing, and lyophilization, the corresponding treated membranes were designated Tr-C and Tr-L. Gross photographs showed that all specimens retained an intact membrane-like morphology before and after treatment. Distinct parallel textures were visible on the surfaces of the longitudinal-cut specimens, whereas no obvious directional texture was observed on the cross-cut specimens.

**FIGURE 1:**
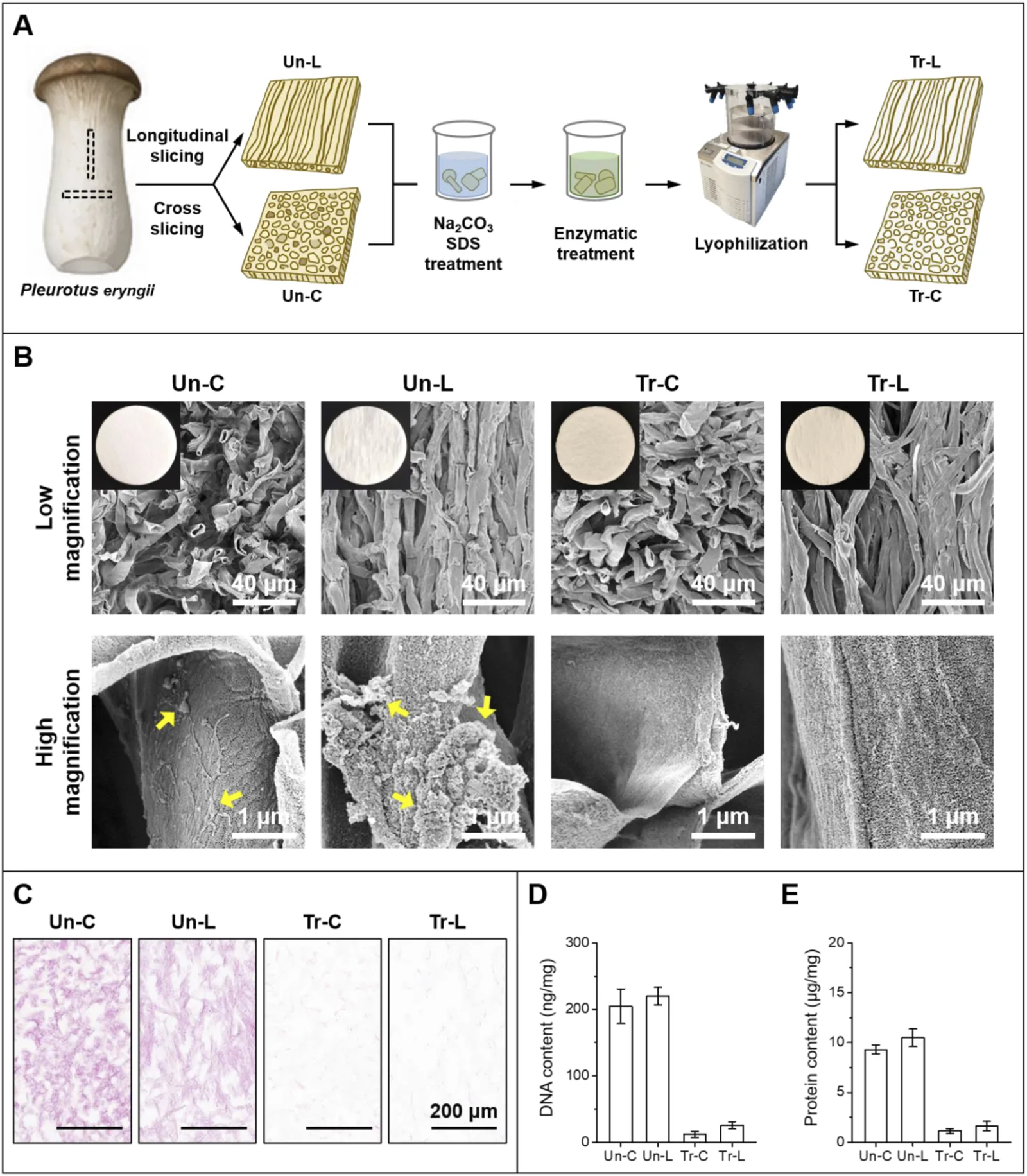
Preparation, morphology, and residual cellular components of Pleurotus eryngii-derived membranes before and after treatment. **(A)** Schematic illustration of cross-cut and longitudinal-cut sectioning of the *P. eryngii* stipe, followed by combined chemical-enzymatic treatment, washing, and lyophilization to prepare the membranes. **(B)** Representative gross photographs and scanning electron microscopy (SEM) images of Un-C, Un-L, Tr-C, and Tr-L. Yellow arrows indicate particulate or amorphous deposits on the hyphal surfaces of untreated specimens. **(C)** Representative H&E-stained sections of Un-C, Un-L, Tr-C, and Tr-L. **(D,E)** Quantification of residual DNA and protein contents. Data are presented as mean ± SD from six independent specimens ( n=6 ).

SEM observations revealed a tubular hyphal network in the untreated specimens (Figure 1B). On the cross-cut surfaces, numerous transverse and oblique hyphal profiles, together with tubular structures oriented in different directions, produced a relatively disordered two-dimensional morphology. In contrast, the tubular hyphal structures on longitudinal-cut surfaces were predominantly aligned in a common direction and exhibited a clear anisotropic organization. After treatment, the original tubular architecture remained readily recognizable in both cutting orientations, without evident overall collapse or large-scale structural disruption, and the orientation characteristics associated with the cutting direction were largely preserved. At higher magnification, the inner and outer surfaces of untreated hyphae were covered with particulate, flocculent, or amorphous deposits. These deposits were markedly reduced after treatment, resulting in more clearly defined hyphal wall contours.

H&E staining further revealed pronounced differences before and after treatment (Figure 1C). Untreated specimens showed relatively intense hematoxylin and eosin staining, whereas staining was markedly attenuated after treatment, leaving only faint outlines of the hyphal walls and surrounding matrix. The orientation characteristics observed in the histological sections were generally consistent with those identified by SEM. Quantification of residual DNA and protein further supported the histological observations. The DNA content of the untreated specimens was approximately 170–220 ng/mg, whereas that of both treated groups decreased to below 30 ng/mg, corresponding to a reduction of approximately 82%–86% (Figure 1D). Similarly, the protein content decreased from approximately 10 μg/mg in the untreated specimens to below 2.5 μg/mg after treatment, representing a removal efficiency of >75% (Figure 1E).

Overall, the combined chemical-enzymatic treatment substantially reduced residual DNA and protein in the *P. eryngii* tissue while largely preserving the native porous tubular hyphal architecture and the structural differences imposed by the cutting orientation. Importantly, Tr-C and Tr-L were derived from the same type of stipe tissue and underwent identical processing, yet retained distinctly different structural presentations: Tr-C predominantly exhibited transverse and oblique hyphal profiles, whereas Tr-L was characterized by tubular hyphal wall units aligned along the membrane plane. Thus, subsequent differences between the two membranes could be interpreted primarily within a framework of similar material composition but different presentations of the native architecture. These findings also indicate that the treatment reduced cell-associated residues while retaining the microstructural basis necessary for further utilization of the intrinsic hyphal architecture of *P. eryngii* (Sun et al., 2022; Kim et al., 2025).

### 3.2 Chemical characteristics, wet-state mechanical properties, and fluid transport of *P. eryngii*-derived membranes with different cutting orientations

ATR-FTIR analysis showed that specimens with different cutting orientations exhibited generally similar spectral profiles under the same treatment condition (Figure 2A). Differences between untreated and treated specimens were limited above 2000 cm^−1^ and were mainly concentrated within the 2000–800 cm^−1^ region. Near the amide I band at approximately 1640 cm^−1^, both Tr-C and Tr-L exhibited reduced absorbance compared with the untreated specimens. Based on the treatment procedure and the preceding results, this change may be mainly associated with the reduction in protein components. Within the 1500–1200 cm^−1^ region, the spectral profiles of the treated specimens shifted toward that of the chitin reference, and similar changes were observed near 1100 and 925 cm^−1^, suggesting an increased relative contribution of chitin after treatment. Meanwhile, the characteristic absorption associated with β-glucan near 848 cm^−1^ was reduced in the treated specimens, suggesting a decrease in its relative contribution.

**FIGURE 2:**
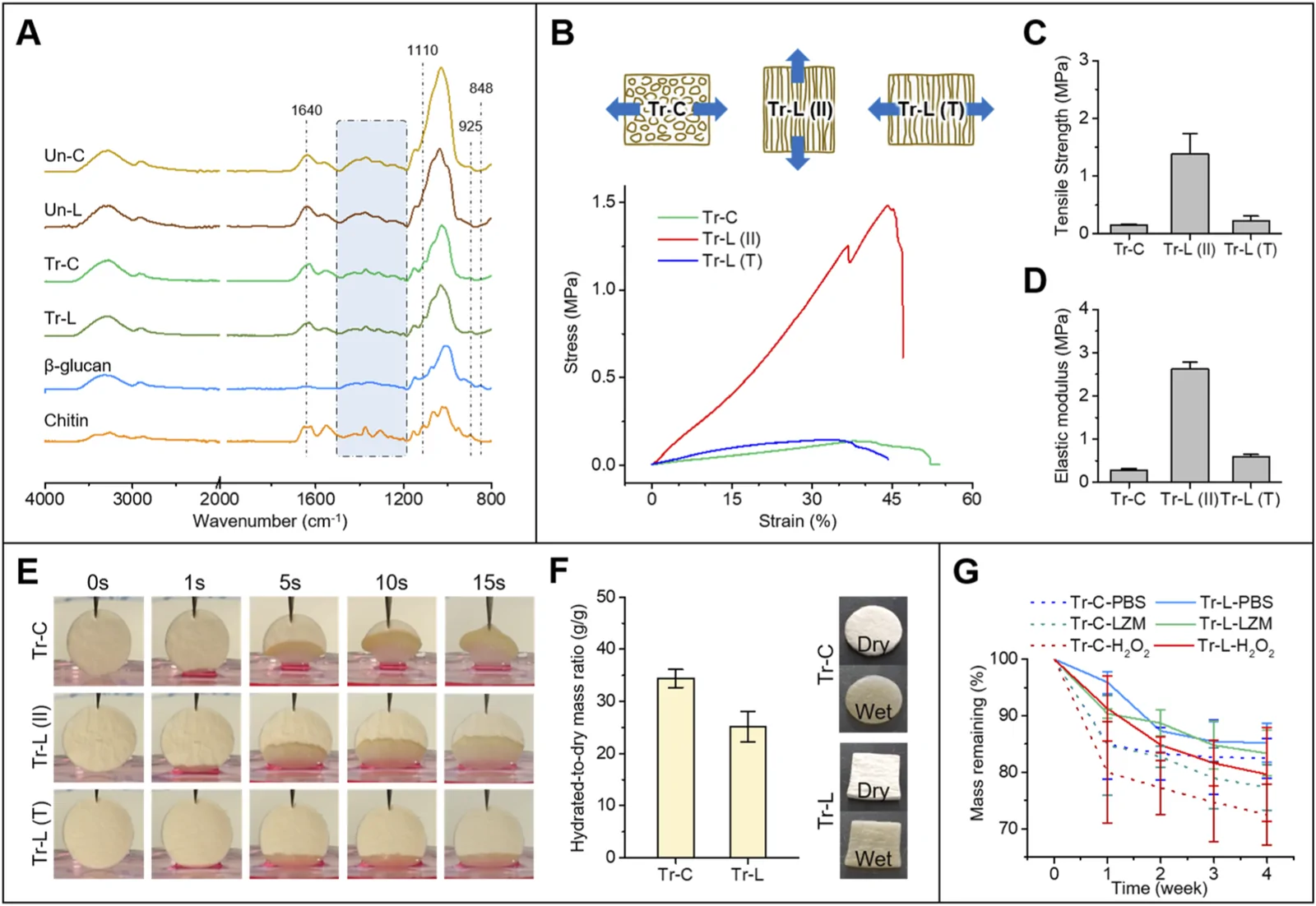
Chemical characteristics, wet-state mechanical properties, liquid infiltration and hydration, and *in vitro* mass changes of *Pleurotus eryngii*-derived membranes. **(A)** ATR-FTIR spectra of the different specimens. **(B)** Wet-state uniaxial tensile testing of Tr-C and Tr-L; the schematic illustrations above indicate the different specimens and loading orientations. **(C,D)** Quantitative analysis of tensile strength and elastic modulus. **(E)** Representative images showing dynamic liquid infiltration into Tr-C and Tr-L membranes, with the liquid front migrating upward from the lower edge of the membrane. **(F)** Quantitative hydrated-to-dry mass ratio, together with representative gross photographs of Tr-C and Tr-L in the dry and fully hydrated states. **(G)** Remaining mass of Tr-C and Tr-L during 4 weeks of incubation in PBS, PBS containing 50 μg/mL lysozyme, or PBS containing 200 μM H_2_O_2_. Data are presented as mean ± SD ( n=4 independent specimens).

To determine how the structural differences generated by cutting orientation affected wet-state mechanical properties, Tr-C and Tr-L membranes were subjected to uniaxial tensile testing (Figure 2B). For Tr-L, loading was applied either parallel or perpendicular to the principal hyphal orientation, designated Tr-L (II) and Tr-L(T), respectively. Representative stress-strain curves showed that Tr-L (II) sustained substantially higher stress throughout tensile loading than Tr-L(T) and Tr-C, whereas the curves of Tr-L(T) and Tr-C were generally similar. Quantitative analysis showed that the tensile strength of Tr-L (II) was approximately 1.48 MPa, markedly higher than those of Tr-L(T) (∼0.19 MPa) and Tr-C (∼0.15 MPa) (Figure 2C). Similarly, the elastic modulus of Tr-L (II) was approximately 3.0 MPa, compared with approximately 0.54 MPa for Tr-L(T) and 0.33 MPa for Tr-C (Figure 2D). These results demonstrate a pronounced orientation-dependent mechanical response of Tr-L under wet conditions.

The liquid infiltration behaviors of Tr-C and Tr-L were further compared, including both Tr-L (II) and Tr-L(T) (Figure 2E). Membranes absorbed the culture medium, but the infiltration behavior showed clear orientation dependence. For Tr-L, liquid migration along the principal hyphal orientation was faster than that perpendicular to the orientation. Tr-L (II) exhibited progressive upward migration of the liquid front, whereas Tr-L(T) showed slower infiltration under the same conditions. Compared with Tr-L (II), Tr-C showed faster infiltration, consistent with its greater exposure of transverse and oblique hyphal profiles and more accessible structural openings.

Hydration measurements further showed that both treated membranes had a high capacity for water absorption (Figure 2F). The hydrated-to-dry mass ratio was approximately 34 g/g for Tr-C and 25 g/g for Tr-L, indicating that the two cutting orientations resulted in different capacities for liquid uptake and retention.

Finally, the *in vitro* mass changes of Tr-C and Tr-L were monitored for 4 weeks in PBS, lysozyme-containing PBS, and H_2_O_2_-containing PBS (Figure 2G). The remaining mass of both membranes gradually decreased over time, although most of the initial mass was retained throughout the experimental period. After 4 weeks, the remaining masses of Tr-L and Tr-C were approximately 85.2% and 82.5%, respectively, in PBS; 83.4% and 77.4% in the presence of lysozyme; and 79.6% and 71.4% under H_2_O_2_ conditions. Across all three media, Tr-C generally exhibited a lower remaining mass than Tr-L, with the greatest mass loss observed under H_2_O_2_ conditions. Notably, Tr-C showed a relatively pronounced decrease in mass during the first week in all three media, followed by a slower rate of mass loss over the subsequent 3 weeks. This early decrease may also include the release of soluble or loosely associated components and the detachment of small structural fragments. Therefore, these measurements are more appropriately interpreted as apparent mass loss/*in vitro* erosion rather than as a direct measure of a specific chemical degradation process.

Overall, Tr-C and Tr-L exhibited similar major chemical characteristics but clear orientation-related differences in wet-state mechanics, liquid infiltration, water uptake, and *in vitro* mass change. Tr-L displayed substantially higher tensile strength and elastic modulus when loaded along the principal hyphal orientation, suggesting that tubular hyphal wall units aligned with the loading direction, together with their interconnected network, can provide longer and more continuous load-transfer pathways. When loading was applied perpendicular to the hyphal orientation, however, the mechanical response approached that of Tr-C, further demonstrating the directional dependence of the retained architecture (Ogawa et al., 2012; Akromah et al., 2026). By contrast, Tr-C exhibited faster liquid infiltration, greater hydration capacity, and greater *in vitro* mass loss. Interestingly, Tr-L itself exhibited transport anisotropy, with faster liquid infiltration along the longitudinal hyphal orientation than perpendicular to it. Together with the orientation-dependent tensile behavior, these results indicate that the preserved hyphal architecture functions as a directional physical framework that regulates both load transfer and fluid migration. These differences may be associated with the greater exposure of accessible luminal and wall openings by transverse and oblique tubular hyphal units, together with shorter or more accessible segments within the discontinuous through-thickness porous network. Given the similar overall ATR-FTIR profiles of the two treated membranes, these functional differences are unlikely to arise from major differences in bulk chemical composition. Nevertheless, different cutting orientations may also alter the surface exposure and accessibility of cell-wall components. Thus, the observed differences should be interpreted primarily within a framework of distinct structural organization and interfacial presentation rather than topography alone. Accordingly, the two cutting orientations did not simply generate membranes with “better” or “worse” properties; rather, they presented the same native hyphal network in distinct configurations that expressed different membrane-scale mechanical, transport, and stability-related functions.

### 3.3 Effects of Tr-C and Tr-L on inflammatory responses of RAW264.7 macrophages

To evaluate the effects of the treated *P. eryngii*-derived membranes on macrophage inflammatory responses, RAW264.7 cells were cultured on Control, Tr-C, and Tr-L substrates under either unstimulated [LPS(-)] or LPS-stimulated [LPS(+)] conditions. Immunofluorescence imaging after 24 h showed that both Tr-C and Tr-L exhibited pronounced intrinsic green autofluorescence, with Tr-L displaying clear parallel directional textures consistent with the SEM and H&E observations. In the absence of LPS, cells adhered to all substrates and tended to form clustered, island-like distributions. After LPS stimulation, the cells became more dispersed, and cell-associated iNOS-positive staining became more apparent relative to the corresponding LPS(-) conditions. Qualitatively, the contrast between cell-associated iNOS staining and the local substrate background appeared more pronounced in the Control group than in the membrane groups, whereas in Tr-L the cell-associated signal was only weakly distinguishable from the intrinsic substrate fluorescence (Figure 3A).

**FIGURE 3:**
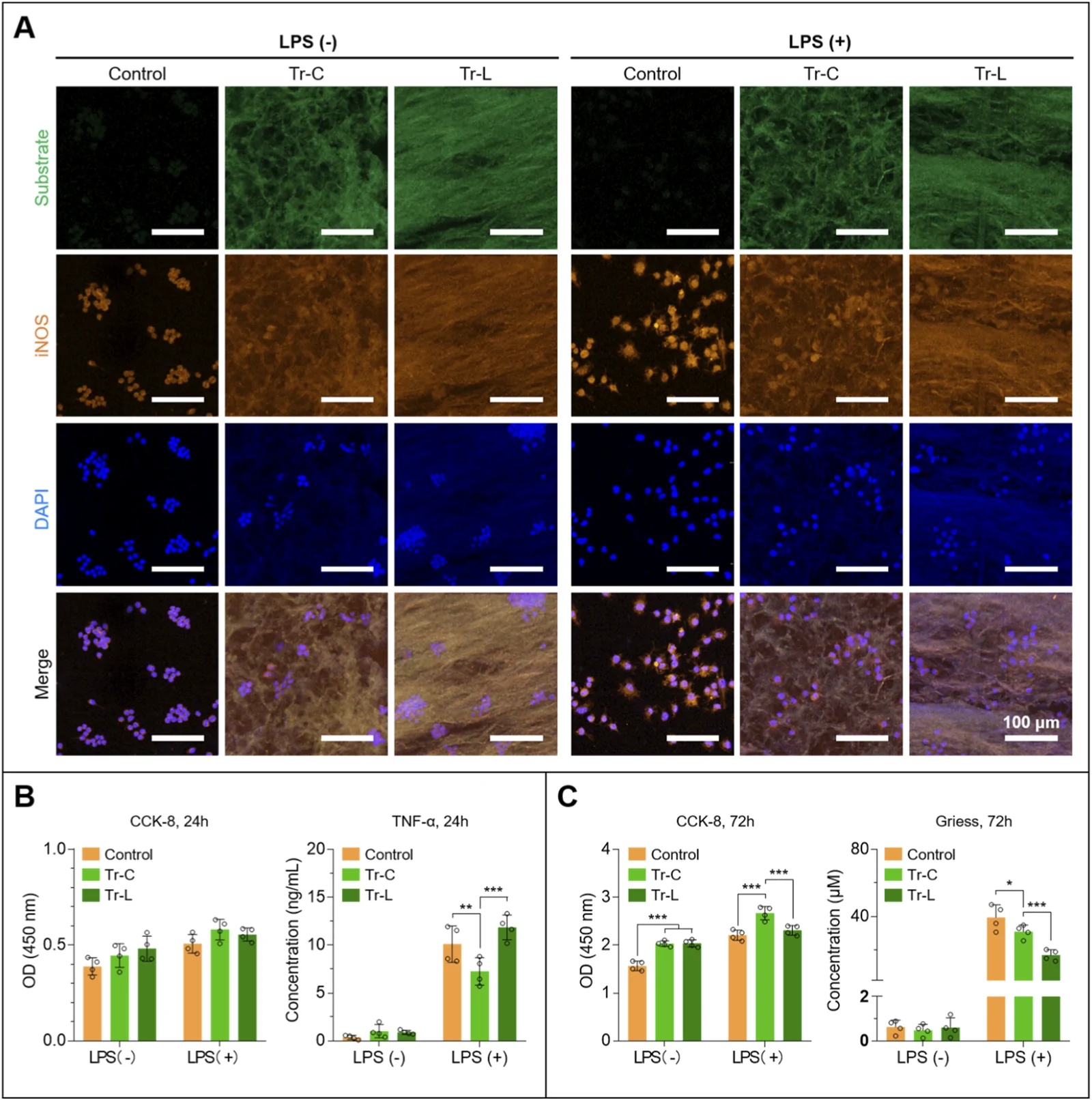
Effects of Tr-C and Tr-L on RAW264.7 macrophage inflammatory responses under basal and LPS-stimulated conditions. **(A)** Representative iNOS immunofluorescence images of RAW264.7 macrophages cultured on Control, Tr-C, and Tr-L for 24 h under LPS(-) and LPS(+) conditions. Green represents intrinsic fluorescence from the membrane substrates, orange indicates iNOS, and blue indicates DAPI-stained nuclei. Scale bar = 100 μm. **(B)** CCK-8 results and TNF-α concentrations in culture supernatants after 24 h **(C)** CCK-8 results after 72 h and nitrite concentrations in culture supernatants measured using the Griess assay. Control, Tr-C, and Tr-L represent the blank culture substrate, treated cross-cut membrane, and treated longitudinal-cut membrane, respectively. Data are presented as mean ± SD ( n=4 independent samples). Statistical analysis was performed by two-way ANOVA; * p<0.05 , ** p<0.01 , *** p<0.001 .

To quantitatively assess macrophage inflammatory responses, TNF-α concentrations in the culture supernatants were measured after 24 h by ELISA, while nitrite concentrations were determined after 72 h using the Griess assay. Because the porous mushroom-derived membranes interfered with conventional total-protein quantification, TNF-α and nitrite levels were not normalized to BCA-derived protein content. Instead, CCK-8 measurements were performed in parallel to determine whether differences in inflammatory output were accompanied by marked changes in cellular metabolic activity.

At 24 h, the mean CCK-8 OD values under LPS(-) conditions were approximately 0.39, 0.45, and 0.48 for Control, Tr-C, and Tr-L, respectively, and increased to approximately 0.51, 0.58, and 0.56 after LPS stimulation (Figure 3B). No significant differences were observed between the membrane groups and the Control, indicating that all groups maintained comparable cellular metabolic activity. Under LPS(-) conditions, TNF-α levels remained low in all three groups, with no significant differences among them. Following LPS stimulation, TNF-α levels increased markedly to approximately 10.10, 7.26, and 11.85 ng/mL in the Control, Tr-C, and Tr-L groups, respectively. The TNF-α level in Tr-C was significantly lower than those in both the Control and Tr-L groups, whereas no significant difference was observed between Tr-L and the Control.

After 72 h of culture, CCK-8 OD values increased further in all groups (Figure 3C). Under LPS(−) conditions, the values were approximately 1.57, 2.03, and 2.04 for Control, Tr-C, and Tr-L, respectively, whereas under LPS(+) conditions they were approximately 2.21, 2.67, and 2.31. These results indicate that neither Tr-C nor Tr-L reduced macrophage metabolic activity during the longer culture period, and under some conditions the OD values were higher than those of the Control.

Nitrite concentrations in the 72-h culture supernatants were then measured as an indicator of NO-related inflammatory output. Under LPS(-) conditions, nitrite levels remained below 1 μM in all three groups, with only minor differences among them. Following LPS stimulation, nitrite concentrations increased to approximately 39.5, 31.0, and 17.0 μM in the Control, Tr-C, and Tr-L groups, respectively. Tr-L exhibited a significantly lower nitrite level than both the Control and Tr-C groups, whereas the difference between Tr-C and the Control was comparatively small.

Taken together, both Tr-C and Tr-L supported RAW264.7 macrophage metabolic activity under basal and LPS-stimulated conditions and did not induce obvious elevations in TNF-α or nitrite in the absence of LPS, suggesting that neither membrane elicited a pronounced sustained pro-inflammatory response on its own. Under LPS stimulation, however, the two membranes exhibited distinct inflammatory output profiles: Tr-C reduced TNF-α levels at 24 h, whereas Tr-L did not differ significantly from the Control; by 72 h, Tr-L showed markedly lower nitrite accumulation than both the Control and Tr-C groups. Although the iNOS images were used only qualitatively because of substrate autofluorescence, the less apparent cell-associated iNOS staining relative to the local substrate background, particularly in Tr-L, was directionally consistent with the lower nitrite accumulation detected at 72 h. This agreement should be interpreted cautiously because the two assays were performed at different time points and reflect different inflammatory readouts. Given that Tr-C and Tr-L were derived from the same tissue source, underwent identical processing, and exhibited similar overall chemical characteristics, these differences may be more closely associated with the distinct hyphal topographies generated by the cutting orientations and the resulting cell-material interfaces, potentially including differences in the exposure and accessibility of cell-wall components at different cut surfaces. Because TNF-α and iNOS/NO represent different inflammatory outputs with distinct temporal dynamics, these findings are more appropriately interpreted as time- and marker-dependent modulation of macrophage inflammatory responses by the two structurally distinct membranes, rather than as a generalized anti-inflammatory effect or an M1-to-M2 polarization transition.

### 3.4 Effects of Tr-C and Tr-L surface structures on MC3T3-E1 cell morphology and orientation

To evaluate the effects of membrane surface structures generated by different cutting orientations on osteogenic cell behavior, MC3T3-E1 cells were cultured on Control, Tr-C, and Tr-L substrates, and cell morphology and alignment were examined by F-actin staining (Figure 4A). On the Control surface, cells spread well but showed a relatively dispersed overall orientation. On Tr-C, cells adhered and spread, with some displaying spindle-shaped or elongated morphologies, but no obvious overall alignment was observed among different cells. In contrast, most cells on Tr-L extended along the parallel surface textures of the membrane and exhibited more pronounced spindle-like morphologies and consistent alignment. Higher-magnification confocal images further showed that F-actin bundles in cells on Tr-L were predominantly distributed along the cellular long axis and exhibited an orientation similar to that of the underlying directional structure.

**FIGURE 4:**
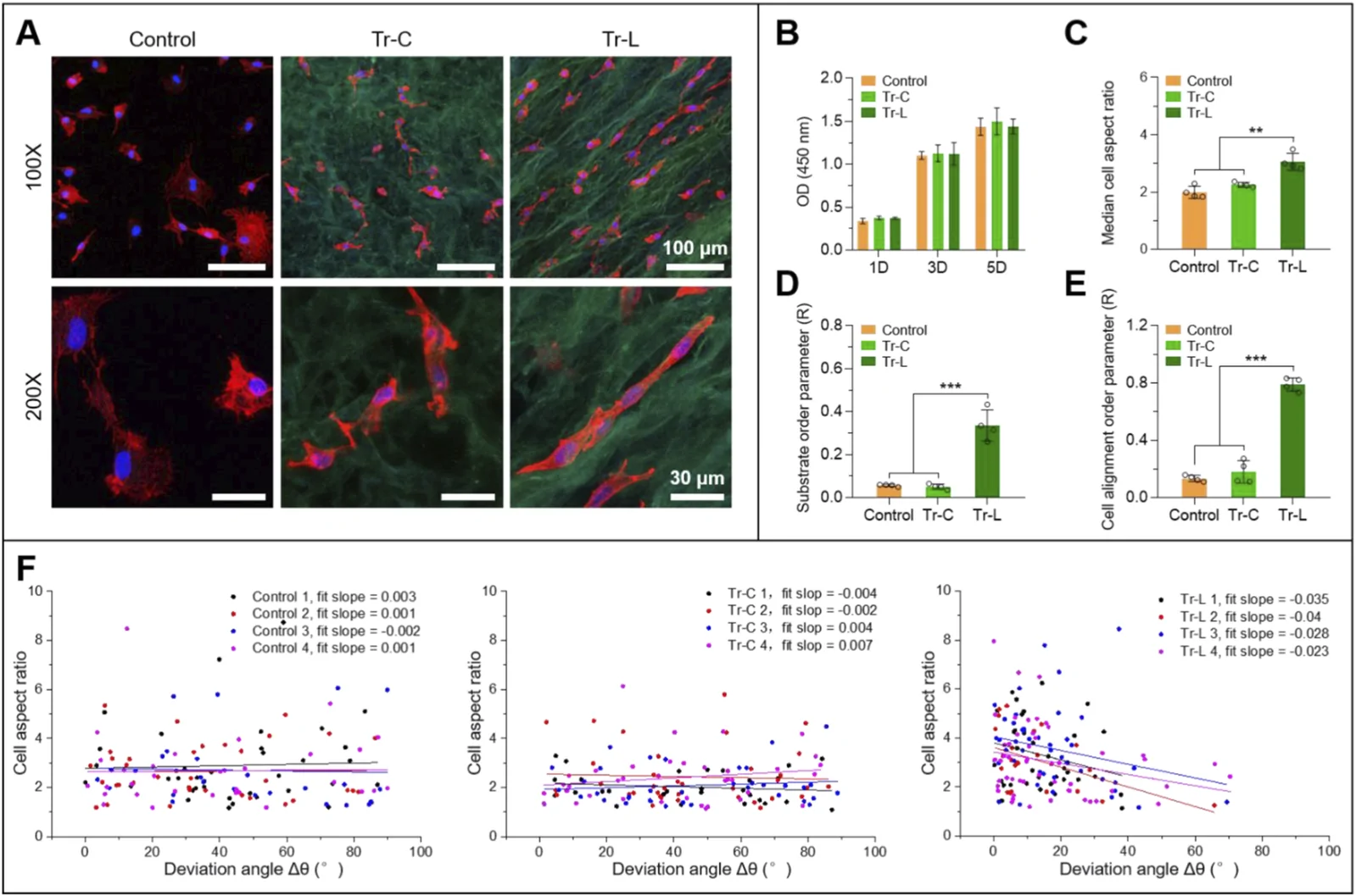
Effects of Tr-C and Tr-L surface structures on MC3T3-E1 cell morphology, metabolic activity, and orientation. **(A)** Representative confocal fluorescence images of MC3T3-E1 cells cultured on Control, Tr-C, and Tr-L. Red indicates F-actin, blue indicates DAPI-stained nuclei, and green represents intrinsic autofluorescence from the membrane substrates. **(B)** CCK-8 results of MC3T3-E1 cells cultured on Control, Tr-C, and Tr-L for 1, 3, and 5 days. **(C)** Quantitative analysis of the median cell aspect ratio in each group. **(D)** Quantitative analysis of the substrate orientation order parameter for Control, Tr-C, and Tr-L. **(E)** Quantitative analysis of the cellular orientation order parameter in each group. **(F)** Relationship between single-cell aspect ratio and the deviation angle between the cellular major axis and the principal substrate orientation in the Control, Tr-C, and Tr-L groups. Different colors indicate independent samples and their corresponding fitted lines. Data are presented as mean ± SD ( n=4 independent samples). For group-level inferential statistics, n represents independent specimens rather than individual cells. Statistical analysis was performed using two-way ANOVA for **(B)** and one-way ANOVA for **(C–E)**; * p<0.05 , ** p<0.01 , *** p<0.001 .

Quantitative analysis showed that the CCK-8 absorbance values increased progressively with culture time in all groups at days 1, 3, and 5 (Figure 4B). No obvious differences were observed among the Control, Tr-C, and Tr-L groups at any time point, indicating that both treated membranes supported sustained MC3T3-E1 cell metabolic activity. The median cell aspect ratio was approximately 2 in both the Control and Tr-C groups, with no significant difference between them. In contrast, the median cell aspect ratio on Tr-L increased to above 3 and was significantly higher than those of the Control and Tr-C groups, indicating a more elongated cell morphology on the Tr-L surface (Figure 4C).

Quantitative analysis of substrate directionality and cellular orientation showed that the substrate orientation order parameter was low in both the Control and Tr-C groups, with values of approximately 0.05. In contrast, the orientation order parameter of Tr-L increased markedly, with an average value above 0.30, indicating a more pronounced structural orientation in Tr-L (Figure 4D). Similarly, the cellular orientation order parameter remained below 0.2 in both the Control and Tr-C groups, with no significant difference between them, whereas the value in the Tr-L group increased significantly to approximately 0.8, indicating highly consistent directional alignment of cells on Tr-L.

Because the substrate orientation order parameters of the Control and Tr-C groups were close to zero, their calculated principal orientations were not robustly defined. Accordingly, the subsequent Δθ analyses for these two groups were regarded mainly as descriptive references, whereas the Tr-L analysis was considered more directly interpretable because of its clearly defined principal structural orientation.

Finally, the relationship between cell elongation and orientation deviation was analyzed by plotting the deviation angle ( Δθ ) between the major axis of each individual cell and the principal orientation of the corresponding substrate against the cell aspect ratio (Figure 4F). In the Control and Tr-C groups, the fitted trends were relatively weak and varied among independent samples. In contrast, all independent Tr-L samples showed a tendency for cell aspect ratio to decrease as Δθ increased, indicating that cells more closely aligned with the principal substrate orientation generally exhibited higher aspect ratios.

Overall, both Tr-C and Tr-L showed good compatibility with MC3T3-E1 cells, but the two membranes produced clear differences in cell morphology and orientation. Tr-L exhibited a significantly higher substrate orientation order parameter, together with a higher cell aspect ratio and a cellular orientation order parameter approaching 0.8. Single-cell analysis further showed that cells more closely aligned with the principal substrate orientation generally exhibited greater elongation. The corresponding changes in substrate and cellular orientation indicate that tubular hyphal units aligned over long distances along the membrane plane can act as stable contact-guidance cues and transmit the directional information of the native architecture to the cellular population scale. In contrast, although Tr-C also exhibited a rough and porous morphology, both its substrate and cellular orientations remained close to random, suggesting that surface roughness or porosity alone is insufficient to generate stable population-level alignment, whereas structural information with long-range directional consistency may be more important. Because no obvious differences in CCK-8 results were observed among the groups, the differences in cell orientation cannot simply be attributed to differences in overall cellular metabolic activity, but instead primarily reflect the contact-guidance effects of the different hyphal spatial architectures on cell morphology and alignment (Zhu et al., 2019; Ogawa et al., 2012; Je et al., 2019).

### 3.5 Effects of Tr-C and Tr-L on osteogenesis-related phenotypes and fibroblast transmembrane migration

To evaluate the effects of the structurally distinct membranes on osteogenesis-related phenotypes of MC3T3-E1 cells, alkaline phosphatase (ALP) staining and activity assays were first performed after 14 days of osteogenic induction (Figures 5A,B). The Control group exhibited relatively uniform but overall weak ALP-positive staining. Staining was enhanced in the Tr-C group, with more dispersed dark-purple regions, whereas Tr-L showed more pronounced ALP-positive staining, with some stained regions distributed along the longitudinal surface textures of the membrane. Quantitative ALP activity measurements were generally consistent with the staining observations. Although the mean values followed the trend Tr-L > Tr-C > Control, relatively large within-group variation was observed in both Tr-L and Tr-C, and only Tr-L was significantly higher than the Control.

**FIGURE 5:**
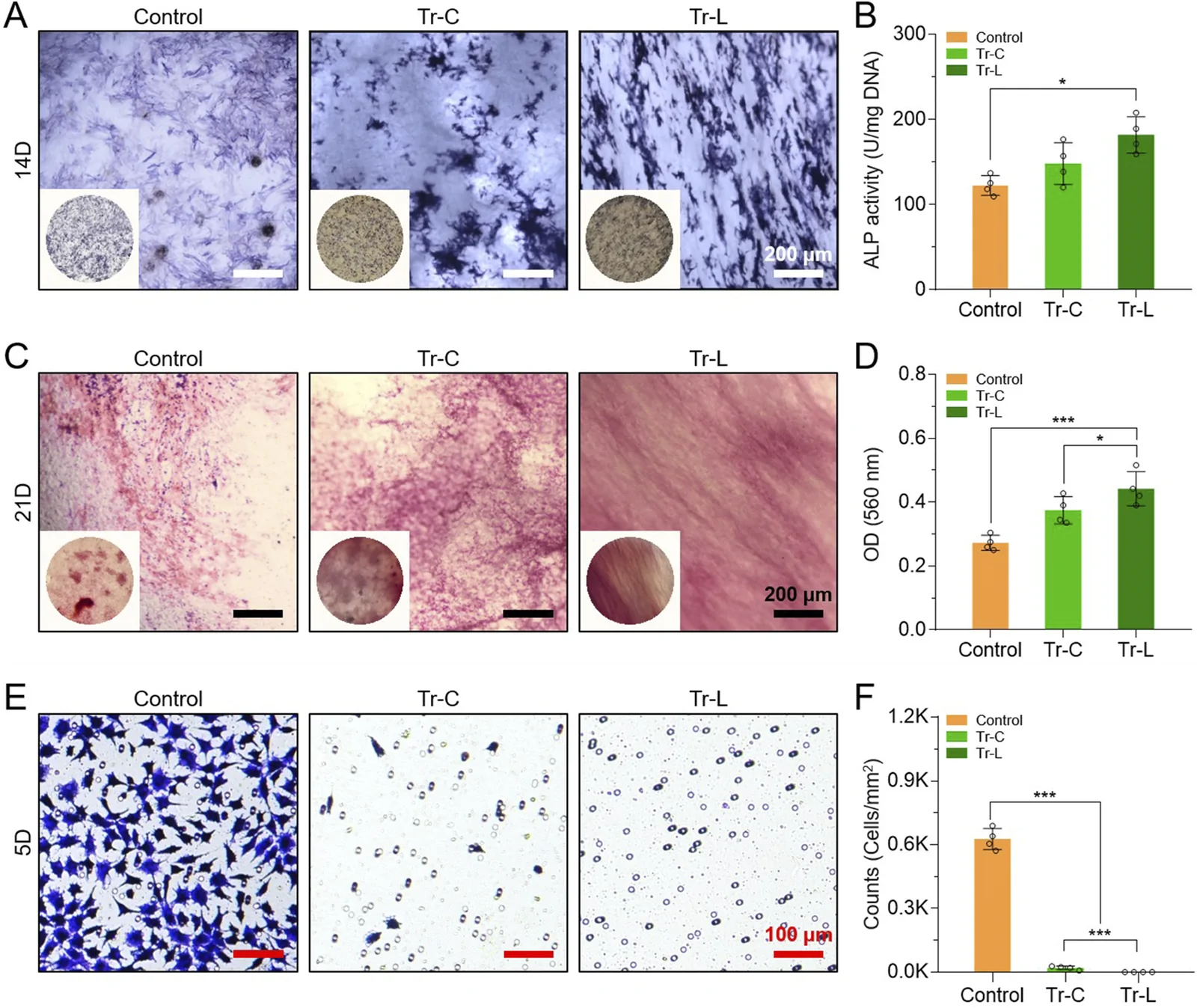
Effects of Tr-C and Tr-L on osteogenesis-related phenotypes of MC3T3-E1 cells and fibroblast transmembrane migration. **(A)** Representative ALP staining images of MC3T3-E1 cells after 14 days of osteogenic induction. **(B)** Quantitative analysis of ALP activity. **(C)** Representative Alizarin Red S (ARS) staining images of MC3T3-E1 cells after 21 days of osteogenic induction. **(D)** Quantification of extracted ARS by absorbance measurement at 560 nm. **(E)** Transwell assay evaluating the ability of the different membranes to restrict fibroblast transmembrane migration. Representative images show fibroblasts that migrated to the lower surface after 5 days of culture. The Control represents the uncovered Transwell membrane, whereas Tr-C and Tr-L represent Transwell inserts covered with the corresponding treated membranes. **(F)** Quantification of migrated fibroblasts. Data are presented as mean ± SD ( n=4 independent samples). Statistical analysis was performed using one-way ANOVA; * p<0.05 , ** p<0.01 , *** p<0.001 .

Alizarin Red S (ARS) staining was subsequently performed after 21 days of osteogenic induction to evaluate a mineralization-related phenotype (Figures 5C,D). Compared with the Control, both Tr-C and Tr-L showed more intense red staining, and Tr-L exhibited a more apparent directional staining pattern. Quantification of the extracted ARS by absorbance measurement at 560 nm showed an overall trend of Tr-L > Tr-C > Control. Tr-L was significantly higher than both Tr-C and the Control, whereas the difference between Tr-C and the Control did not reach statistical significance.

The ability of Tr-C and Tr-L to restrict fibroblast transmembrane migration was further evaluated using a Transwell system (Figures 5E,F). In the Control group, in which the Transwell membrane was left uncovered, a large number of migrated cells were observed on the lower surface after 5 days of culture and were densely distributed throughout the field of view. In contrast, substantially fewer migrated cells were observed in the Tr-C group, with only scattered cells present on the lower surface, whereas no migrated cells were observed in the Tr-L group. Quantitative cell counting showed that both Tr-C and Tr-L significantly reduced fibroblast migration compared with the Control, with Tr-L further showing a significant reduction compared with Tr-C.

Overall, both Tr-C and Tr-L exhibited more pronounced osteogenesis-related phenotypes than the Control and significantly restricted fibroblast transmembrane migration, with Tr-L showing the most prominent effects in ARS quantification and fibroblast exclusion. Combined with the preceding cell-orientation results, the tubular hyphal units in Tr-L, which were aligned over long distances along the membrane plane, not only provided directional contact-guidance cues for MC3T3-E1 cells but may also have created a structural interface favorable for the subsequent development of osteogenesis-related phenotypes. Meanwhile, because most tubular hyphal units in Tr-L were oriented primarily along the membrane plane, fewer transverse profiles and structural openings were directly exposed in the through-thickness direction, and fibroblast transmembrane migration may therefore have required more tortuous and discontinuous structural pathways. By contrast, Tr-C exposed more transverse and oblique hyphal profiles and luminal openings, which was consistent with its faster liquid infiltration and relatively weaker cell-barrier performance. Thus, the structural advantage of Tr-L did not simply arise from being “denser” or having “fewer pores,” but rather from an orientation-dependent functional division: providing cell guidance within the membrane plane while forming a soft-tissue barrier across the membrane thickness. This combination of in-plane cell guidance and through-thickness fibroblast exclusion is well aligned with the dual requirements of GBR membranes for cellular regulation and spatial isolation.

### 3.6 Tr-C and Tr-L promote centripetal bone regeneration in rat calvarial defects

To evaluate the *in vivo* bone-regenerative effects of the structurally distinct *P. eryngii*-derived membranes, bilateral 5-mm full-thickness calvarial defects were created in adult SD rats and treated with either Tr-C or Tr-L membranes, with untreated defects serving as the Control. Bone regeneration was evaluated by micro-CT and histological analyses at 6 and 12 weeks after surgery (Figure 6).

**FIGURE 6:**
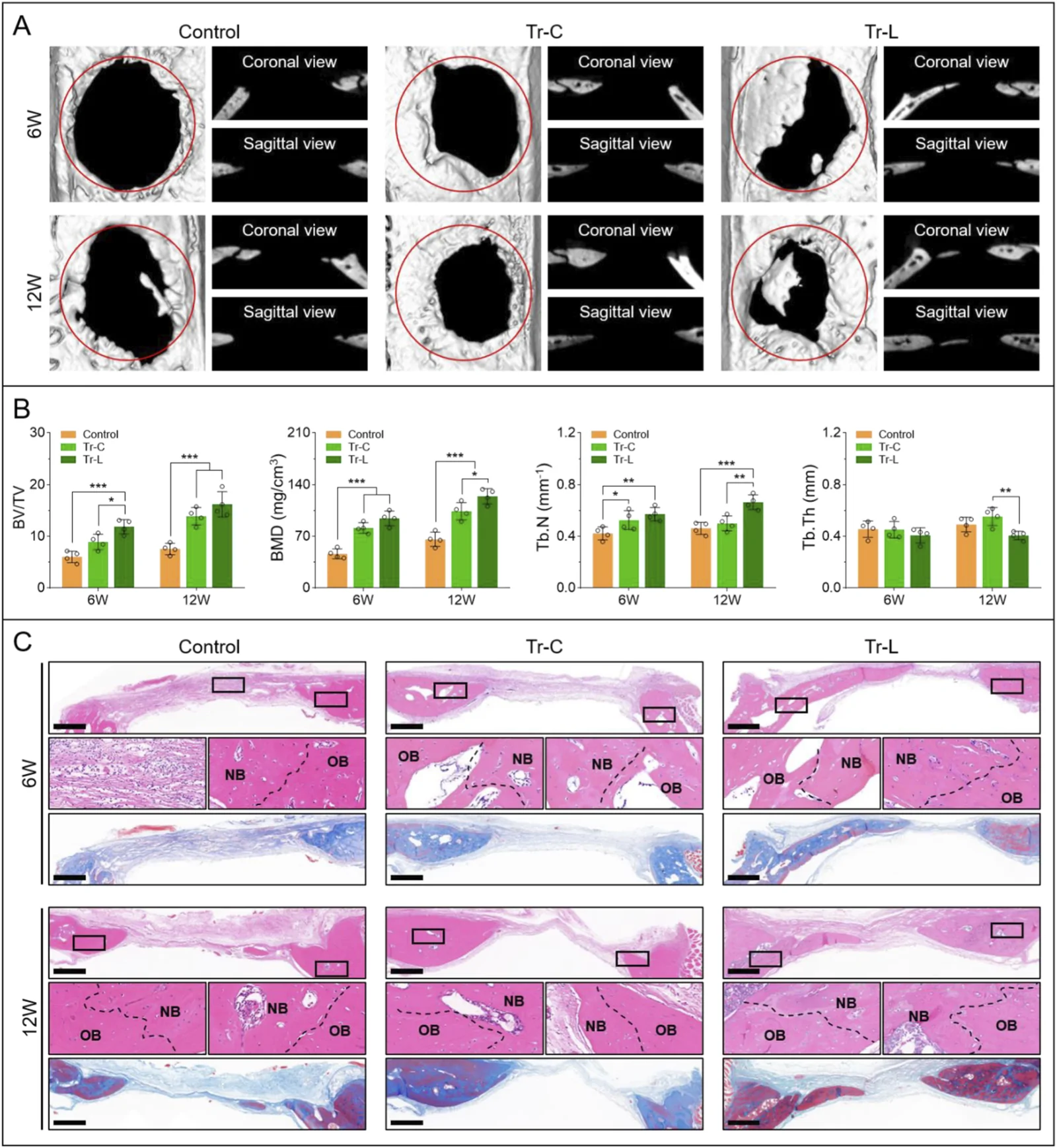
Effects of Tr-C and Tr-L on bone regeneration in rat calvarial defects. **(A)** Representative three-dimensional micro-CT reconstructions and coronal and sagittal sectional images of calvarial defects in the Control, Tr-C, and Tr-L groups at 6 and 12 weeks after surgery. Red circles indicate the original 5-mm defect regions. **(B)** Quantitative micro-CT analysis, including bone volume/total volume (BV/TV), bone mineral density (BMD), trabecular number (Tb.N), and trabecular thickness (Tb.Th). **(C)** Representative histological images of calvarial defects in the three groups at 6 and 12 weeks. The upper panels show low-magnification panoramic H&E-stained sections and corresponding higher-magnification images of the boxed regions, whereas the lower panels show Masson’s trichrome staining, bar = 500 μm. NB indicates newly formed bone and OB indicates old bone; dashed lines indicate the boundary region between newly formed and old bone. Data are presented as mean ± SD, with n=4 defects from 4 different animals per group at each time point. Statistical analysis was performed using one-way ANOVA separately at each time point; * p<0.05 , ** p<0.01 , *** p<0.001 .

Three-dimensional micro-CT reconstruction together with coronal and sagittal sectional images showed that, at 6 weeks, a large non-mineralized region remained in the center of the Control defects, with newly formed bone mainly confined to the original defect margins. In the Tr-C group, more high-density bone-like tissue was observed along the defect edges than in the Control, whereas Tr-L showed a further increase in the extent of newly formed bone, which extended centripetally from the defect margins toward the center (Figure 6A). By 12 weeks, bone formation had increased in all three groups compared with that at 6 weeks, although complete bony bridging was not observed in any group. A clear residual defect remained in the center of the Control group, whereas the residual defect area was reduced in Tr-C. Tr-L showed more extensive new bone formation within the defect and more pronounced centripetal closure.

Quantitative micro-CT analysis further supported these observations (Figure 6B). As the observation period increased from 6 to 12 weeks, bone volume fraction (BV/TV) and bone mineral density (BMD) generally increased in all three groups. At both time points, BV/TV and BMD showed an overall increasing trend from Control to Tr-C to Tr-L, with Tr-L exhibiting the highest values. Trabecular number (Tb.N) showed a similar pattern, particularly at 12 weeks, when Tr-L was higher than both the Control and Tr-C groups. In contrast, the groupwise pattern of trabecular thickness (Tb.Th) was not fully consistent with those of the other parameters. Tr-L did not exhibit a higher Tb.Th at either 6 or 12 weeks, and Tr-C showed a higher Tb.Th than Tr-L at 12 weeks.

Histological findings were generally consistent with the micro-CT results (Figure 6C). At 6 weeks, the defect region in the Control group was predominantly occupied by soft tissue, with new bone mainly located near the margins of the original bone. Both Tr-C and Tr-L showed more newly formed bone extending from the defect margins toward the interior, with the extent of new bone formation being more pronounced in Tr-L. Higher-magnification H&E images showed a continuous transition region between newly formed bone (NB) and old bone (OB).

By 12 weeks, new bone formation had further increased in all groups, although central defects of varying sizes remained and complete bony bridging was still not observed. In the Control group, newly formed bone remained mainly concentrated along the bilateral defect margins. Tr-C showed greater extension of new bone toward the defect center, whereas Tr-L exhibited more extensive new bone tissue and a more pronounced reduction in defect size. Masson’s trichrome staining similarly showed progressive increases in collagenous matrix and bone-like tissue within the defect margins and newly formed tissue regions over time, with both Tr-C and Tr-L showing broader areas of tissue repair than the Control.

Overall, both Tr-C and Tr-L promoted bone regeneration in the 5-mm rat calvarial defect model, with Tr-L showing more pronounced improvements in BV/TV, BMD, Tb.N, and the histological extent of newly formed bone. Although Tr-L did not exhibit the highest value for every micro-CT parameter, as exemplified by the divergence of Tb.Th from BV/TV and Tb.N at some time points, this does not contradict the overall increase in new bone formation. BV/TV, Tb.N, and Tb.Th reflect different aspects of bone quantity and microarchitecture and therefore do not necessarily change in parallel during bone regeneration. Accordingly, the higher BV/TV and Tb.N observed in Tr-L primarily support an increase in the overall amount of mineralized bone and the number of trabecular structures within the defect (Bouxsein et al., 2010).

Taken together, the material, cellular, and animal results suggest that the superior bone-regenerative performance of Tr-L *in vivo* likely arose from the combined contribution of multiple structural and biological functions rather than from any single parameter. In Tr-L, the preserved tubular hyphal units were predominantly aligned along the membrane plane, resulting in pronounced wet-state mechanical anisotropy and a stable, long-range directional topography at the cell-material interface. By contrast, Tr-C exposed more transverse and oblique hyphal profiles and exhibited faster liquid infiltration and greater hydration capacity, but weaker wet-state load-bearing capacity and directional cell-guidance effects. Thus, the two cutting orientations did not simply produce “better” and “worse” materials, but instead translated the same native hyphal network into distinct functional states. Among them, the combination of structural stability, in-plane cell guidance, and through-thickness barrier function provided by Tr-L more closely matched the integrated requirements of GBR membranes for space maintenance and cellular regulation.

The regulation of MC3T3-E1 cell morphology and orientation by Tr-L may represent one factor contributing to its enhanced osteogenesis-related phenotype. Previous studies have shown that cell spreading, aspect ratio, cytoskeletal tension, and directional alignment are not merely morphological features, but can influence osteogenic gene expression and differentiation through mechanosensing and mechanotransduction processes involving integrin/FAK, RhoA/ROCK, and YAP/TAZ signaling (Zhu et al., 2019; Shi et al., 2023). In the present study, Tr-L significantly increased cell aspect ratio and the population-level orientation order parameter and was accompanied by more pronounced ALP- and ARS-related osteogenic phenotypes. These observations are consistent with the established relationship among topographical cues, cytoskeletal reorganization, and osteogenic differentiation. However, because these signaling pathways were not directly examined in this study, the present results only support the interpretation that Tr-L provides a mechanical and morphological microenvironment more favorable for directional cell spreading and osteogenesis-related behavior, rather than demonstrating a specific molecular mechanotransduction mechanism.

Interactions between the membranes and macrophages may also have contributed to the subsequent regenerative response. Under LPS(-) conditions, neither membrane induced a marked sustained increase in TNF-α or NO-related inflammatory output. Following LPS stimulation, Tr-C reduced TNF-α release at 24 h, whereas Tr-L showed a more pronounced reduction in nitrite accumulation at 72 h. Given the similar overall chemical characteristics of the two membranes, these differences may primarily reflect the distinct cell-material interfaces created by different hyphal topographies, together with differences in the exposure and accessibility of native cell-wall components at different structural surfaces. Meanwhile, *P. eryngii*-derived polysaccharides themselves have immunomodulatory potential, and their biological effects are known to depend on molecular structure and presentation state (Ma et al., 2020; Ellefsen et al., 2023). Therefore, the macrophage responses observed here are more appropriately interpreted as structure- and interface-related differences rather than being directly attributed to a specific β-glucan or defined as a clear anti-inflammatory mechanism.

From this perspective, the *in vivo* performance of Tr-L may reflect contributions at several levels: the interconnected hyphal network provides favorable wet-state structural stability; the in-plane directional topography promotes elongation and alignment of osteogenesis-related cells; the more tortuous and discontinuous through-thickness structural pathways restrict fibroblast transmembrane migration; and the native chitin-glucan cell wall and its interactions with immune cells may further influence the local tissue microenvironment. Such a multifactorial contribution may also explain why Tr-L was not superior in every individual material parameter, yet showed better overall outcomes in BV/TV, BMD, Tb.N, and histological new bone formation.

The present study also has limitations in terms of mechanistic depth. First, the analysis of structure-cell relationships remained primarily at the morphological and functional-phenotype levels, and the molecular link among hyphal topography, cell orientation, and osteogenic differentiation was not further established through transcriptomic analysis, qPCR, protein expression, or mechanotransduction pathway studies. Second, FTIR mainly reflects overall chemical characteristics and cannot quantitatively resolve the chitin/β-glucan ratio, β-glucan conformation, or surface exposure state. The relative contributions of native topography and native chemical composition to immune and osteogenic responses therefore remain difficult to distinguish. In addition, although Tr-L significantly promoted centripetal new bone formation, complete bony bridging was not achieved within 12 weeks, suggesting that the unactivated native hyphal membrane is more appropriately considered an architecture-driven GBR platform rather than a strongly osteoinductive material. Previous systematic reviews have also indicated that spontaneous complete closure of 5-mm calvarial defects in adult rats is uncommon and that 5-mm defects can be used as a critical-size defect model (Vajgel et al., 2014). Notably, the preserved tubular hyphal cell walls and multiscale porous architecture not only provide directional mechanical and cell-guidance functions, but also offer potential structural space for subsequent loading of drugs, osteogenic peptides, mineral components, or other bioactive molecules. Future interfacial activation, mineralization, or controlled loading strategies that preserve the native hyphal architecture and directionality, together with further investigation of mechanotransduction and chitin-β-glucan structure-activity relationships, may further enhance bone regeneration while retaining the advantages of in-plane cell guidance and through-thickness barrier function.

## 4 Conclusion

This study demonstrates that the native longitudinal hyphal organization of *Pleurotus eryngii* can be preserved and expressed as spatially differentiated membrane functions. Compared with the cross-sectional architecture, the longitudinally organized hyphal structure provided a favorable combination of in-plane cell guidance and through-thickness barrier function and was associated with enhanced centripetal bone regeneration *in vivo*. These findings highlight naturally preorganized biological architectures as a structural resource for constructing functionally anisotropic GBR membranes without relying on complex artificial fabrication or exogenous bioactive components.

## Data Availability

The original contributions presented in the study are included in the article/supplementary material, further inquiries can be directed to the corresponding authors.
